# Acute Abdominal Pain Secondary to Chilaiditi Syndrome

**DOI:** 10.1155/2013/756590

**Published:** 2013-07-01

**Authors:** David Kang, Andrew S. Pan, Michael A. Lopez, Jessica L. Buicko, Miguel Lopez-Viego

**Affiliations:** ^1^NOVA Southeastern University College of Osteopathic Medicine, Davie, FL, USA; ^2^Department of Surgery, University of Miami, JFK Medical Center, Atlantis, FL, USA; ^3^Department of General and Vascular Surgery, JFK Medical Center, Atlantis, FL, USA; ^4^Bethesda Memorial Hospital, Boynton Beach, FL, USA

## Abstract

Chilaiditi syndrome is a rare condition occurring in 0.025% to 0.28% of the population. In these patients, the colon is displaced and caught between the liver and the right hemidiaphragm. Patients' symptoms can range from asymptomatic to acute intermittent bowel obstruction. Diagnosis is best achieved with CT imaging. Identification of Chilaiditi syndrome is clinically significant as it can lead to many significant complications such as volvulus, perforation, and bowel obstruction. If the patient is symptomatic, treatment is usually conservative. Surgery is rarely indicated with indications including ischemia and failure of resolution with conservative management.

## 1. Introduction

 Chilaiditi sign is defined as the interposition of bowels between the liver and right diaphragm [1]. Chilaiditi sign is also referred to as hemidiaphragmatic interposition of the colon. This sign was first described in the medical literature in 1910 by the Greek radiologist Demetrius Chilaiditi [2]. This condition occurs in 0.25% to 0.28% of the population and typically involves the hepatic flexure or transverse colon [3]. A radiologic finding of hemidiaphragmatic interposition of the colon is referred to as Chilaiditi sign, while a symptomatic case is known as Chilaiditi syndrome. Abdominal pain, constipation, vomiting, respiratory distress, anorexia, volvulus, and obstruction are possible presentations of Chilaiditi syndrome [1]. Chilaiditi syndrome can be a self-resolving or a chronic condition [3]. We present a rare case of a 57-year-old female who presented with epigastric and right upper quadrant pain with radiation to the right shoulder. Initially believed to be biliary colic or musculoskeletal pain, the patient was found to have Chilaiditi syndrome diagnosed by CT scan. 

## 2. Case Presentation

A 57-year-old Caucasian female presented to the emergency department with a 24-hour history of nausea and worsening epigastric and right upper quadrant pain. The pain was sharp in nature and radiated to the right shoulder. She denied emesis, dysphagia, early satiety, fever, chills, night sweats, melena, hematochezia, or any changes in her bowel habits. She did not recall having a similar experience before. Her past medical history included GERD, anxiety, and chronic lower back pain, which was controlled with omeprazole, alprazolam, and ibuprofen, respectively. Her past surgical history included a laminectomy for her back pain. She denied tobacco, alcohol, or illicit drug use. Her family history was significant for a father with hereditary hemochromatosis, mother with diverticulosis and IBS, and grandmother with colon cancer. 

On original presentation, she was afebrile, with a blood pressure of 156/91 mmHg, pulse of 77 beats/min, respiratory rate of 20 beats/min, and oxygen saturation of 99% on room air. On physical examination, the cardiovascular and respiratory exams were unremarkable. Her abdomen was soft but exhibited tenderness to palpation in the epigastrium as well as the right upper quadrant. Murphy's sign was equivocal. There were no signs of rebound tenderness, guarding, or ascites.

**Figure 1:**
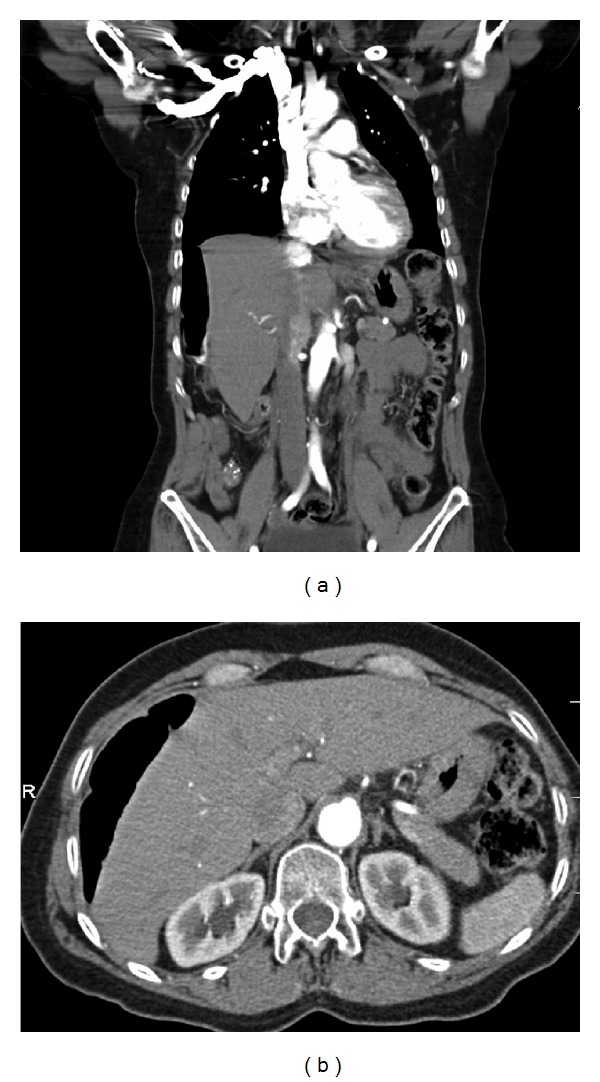
(a) Coronal CT view. (b) Axial CT view. Both demonstrate a loop of colon interpositioned between the liver and right hemidiaphragm.

Basic laboratory studies revealed a mild hyponatremia (134 mmol/dL). A hepatic panel was within normal limits. Complete blood count demonstrated a normal wbc and platelet count accompanied by a mild anemia (Hb/Hct 11.8/35.4). Her cardiac enzymes and urinalysis were unremarkable. Imaging studies showed unremarkable chest X-ray and abdominal ultrasound. A HIDA scan with CCK showed a decreased gallbladder ejection fraction of 33% (normal: 35–75%). Further imaging by CT scan of the thorax, abdomen, and pelvis showed a loop of colon interpositioned between the liver and right hemidiaphragm, mimicking free air (Figure 1). There was mild bowel wall thickening involving the mid-transverse colon and descending colon, but there was no evidence of bowel obstruction. These findings suggested Chilaiditi's syndrome.

The patient was managed conservatively with IV fluid hydration and pain management. During the course of her hospital stay, her abdominal pain resolved without surgical intervention. It was believed that her interrupted large bowel obstruction was no longer intertwined between the liver and right hemidiaphragm. She was then able to tolerate a regular diet and was discharged after four days of hospital stay. 

## 3. Discussion

 Chilaiditi syndrome is extremely rare. With normal human anatomy, the suspensory ligaments of the liver, mesocolon, liver, and the falciform ligament are situated in a manner that minimizes space surrounding the liver and prevents interposition of the colon. A patient is predisposed to Chilaiditi syndrome when there is deviation of the structures surrounding the liver. 

 The etiology of Chilaiditi syndrome can be congenital or acquired. Predisposing congenital abnormalities include absent suspensory or falciform ligaments, redundant colon, malpositions, dolichocolons, and paralysis of the right diaphragm [1, 4]. Acquired risk factors include chronic constipation, cirrhosis leading to liver atrophy, obesity, multiple pregnancies, ascites, and paralysis of the right diaphragm [4]. Men are four times more likely than women to develop Chilaiditi syndrome [3]. Chilaiditi syndrome is most commonly seen in the elderly with a cadence of 1%, but there have been cases where it was presented in patients as young as 5 months [5].

 Chilaiditi syndrome has been known to cause severe complications including volvulus of the cecum, splenic flexure or transverse colon, cecal perforation, and subdiaphragmatic appendicitis perforation [4]. An undiagnosed Chilaiditi's sign increases the risk of perforation during liver biopsy and colonoscopy [4]. In addition, Chilaiditi syndrome has been linked with pulmonary and gastrointestinal malignancies [4].

 In the diagnosis of Chilaiditi sign, the first step is to rule out the possibility of pneumoperitoneum. For the diagnosis to be made by imaging, the right hemidiaphragm must be displaced superiorly to the liver by the intestines, pseudoperitoneum caused by air in the bowels must be seen, and the superior aspect of the liver must be positioned below the level of the left hemidiaphragm. The best imaging modality for visualization is CT scan which carries an added benefit of ruling out the possibility of diaphragmatic rupture. Another indication of Chilaiditi sign is when a patient changes positions, the area of radiolucency will not shift as seen in free air [4]. If Chilaiditi sign is visualized and the patient is symptomatic, it is then referred to as Chilaiditi's syndrome.

 With regard to treatment of Chilaiditi syndrome, conservative management (bed rest, intravenous fluids, nasogastric decompression, enemas, cathartics, high fiber diet, and stool softeners) should be attempted first [1]. If repeat imaging shows failure of resolution or if ischemia is suspected, surgical treatment is indicated. Cecopexy is recommended for an uncomplicated cecal volvulus, while colonic resection is the best option if the volvulus involves the transverse colon. Involvement of the transverse colon carries a high frequency of gangrene; therefore, colonoscopic reduction is not suggested [4]. 

 In conclusion, although a rare condition, Chilaiditi's syndrome has important clinical ramifications. Chilaiditi is a rarely considered differential diagnosis with vague symptoms that make diagnosis difficult. Differentials of Chilaiditi syndrome include bowel obstruction, volvulus, intussusception, ischemic bowel, appendicitis, and diverticulitis [4]. Even after imaging is performed, Chilaiditi can still be misinterpreted as Morgagni's hernia, subdiaphragmatic hernia, and pneumoperitoneum [1]. Kamiyoshihara presents a case where after a traffic accident, a 75 year old is believed to have developed a traumatic diaphragmatic hernia. A CT scan was showed what was believed to be a section of colon that had herniated through the diaphragm. The patient underwent exploratory video-assisted thoracoscopic surgery where it was discovered there had been no injury to the diaphragm or any other organs despite the strong clinical suspicion. The patient was then diagnosed with Chilaiditi sign, which could have been managed conservatively [1]. In our case, the patient was initially believed to have either biliary colic or musculoskeletal pain. But a proper workup and keeping in mind the possibility of Chilaiditi syndrome kept the patient out of the operating room for what would have proven to be an unnecessary procedure.
